# The cGAS–STING pathway: more than fighting against viruses and cancer

**DOI:** 10.1186/s13578-021-00724-z

**Published:** 2021-12-14

**Authors:** Terigen Bao, Jia Liu, Jiyan Leng, Lu Cai

**Affiliations:** 1 Department of Geriatrics, The First Hospital of Jilin University, Changchun, 130021 China; 2 Department of Pediatrics, The Pediatric Research Institute, The University of Louisville School of Medicine, Louisville, KY 40292 USA; 3 Departments of Pharmacology and Toxicology, The University of Louisville School of Medicine, Louisville, KY USA

**Keywords:** Anti-virus inflammation, cGAS–STING pathway, Diabetes, Insulin resistance, Diabetes complications

## Abstract

In the classic Cyclic guanosine monophosphate–adenosine monophosphate (cGAMP) synthase (cGAS)-stimulator of interferon genes (STING) pathway, downstream signals can control the production of type I interferon and nuclear factor kappa-light-chain-enhancer of activated B cells to promote the activation of pro-inflammatory molecules, which are mainly induced during antiviral responses. However, with progress in this area of research, studies focused on autoimmune diseases and chronic inflammatory conditions that may be relevant to cGAS–STING pathways have been conducted. This review mainly highlights the functions of the cGAS–STING pathway in chronic inflammatory diseases. Importantly, the cGAS–STING pathway has a major impact on lipid metabolism. Different research groups have confirmed that the cGAS–STING pathway plays an important role in the chronic inflammatory status in various organs. However, this pathway has not been studied in depth in diabetes and diabetes-related complications. Current research on the cGAS–STING pathway has shown that the targeted therapy of diseases that may be caused by inflammation via the cGAS–STING pathway has promising outcomes.

## Introduction

In the past 20 years, metabolic syndrome (MetS) has evolved from a lesser understood term to a commonplace topic. The MetS has also become a risk factor for many chronic diseases [1]. Evidence confirms that the MetS and inflammation are closely related [1–3], and the link between the MetS and inflammation has provided very interesting ideas about the treatment of the MetS. As for diabetes mellitus (DM), a representative type of MetS, current studies also demonstrate that although the etiology of diabetes is not fully clear, inflammation and autoimmunity contribute significantly to the development of diabetes [4–6], and treatment of the inflammatory response to diabetes has shown beneficial therapeutic effects [7]; therefore, further research on the pathogenesis of diabetes and its complications is necessary.

Inflammatory responses and impaired mitochondrial function have been implicated in the function of many organs, and many of these effects are detrimental. Recently, investigations on the cyclic guanosine monophosphate–adenosine monophosphate (cGAMP) synthase (cGAS)-stimulator of interferon genes (STING) pathway have increased with a focus on its association with innate immunity. Initially, the cGAS–STING pathway was primarily concerned with immune mechanisms in infectious diseases [8, 9]. As research progressed, researchers identified impressive expressions of the cGAS–STING pathway in cancer and autoimmune diseases as well [10–12]. Recently, surprisingly, some work has demonstrated that the cGAS–STING pathway is highly associated with lipid metabolism [13, 14]. Therefore, an investigation of whether the cGAS–STING pathway is participating in and influencing the course of diabetes and its subsequent complications is warranted. Here, we will selectively summarize some literature reports on the effects of the cGAS–STING pathway in different organ diseases and focus on the possible cross-talk between the cGAS–STING pathway and diabetes. Since there is no clearer direct evidence of the link between the cGAS–STING pathway and diabetes, through some indirect literature reports, we can still observe an inextricable link between the cGAS–STING pathway and diabetes and its complications. This review may provide useful insights into the precise treatment of diabetes and may help guide future research.

## The cGAS–STING pathway

STING (transmembrane protein 173, TMEM173 and MPYS/MITA/ERIS) is an evolved consensus transmembrane protein found in the endoplasmic reticulum (ER) of mammals and protozoa [15]. When STING was first discovered in 2008, researchers were investigating open reading frames that induce antiviral gene expression [16, 17]. Coincidentally, some researchers discovered that STING is involved in the transduction of proapoptotic signals [18]. Subsequently, STING was reported to exert antiviral effects through the TANK-binding kinase 1 (TBK1)-interferon regulatory factor 3 (IRF3) axis [16, 17], but the proteins related to its upstream signaling function were not confirmed at that time. Later, discoveries led by Chen confirmed that cGAS is a direct DNA receptor. Upon direct binding of cytoplasmic DNA, cGAS forms a dimer that catalyzes the production of 2′3′-cGAMP from ATP and GTP, and then the binding of cGAMP activates STING, which facilitates the mobilization of downstream molecules [19, 20].

Recent works have also identified several other critical functions of the cGAS–STING pathway, which may also be involved in antiviral mechanisms and other chronic disease responses. For example, the cGAS–STING pathway was demonstrated to have a role in cancer and lipid metabolism as well as in a range of chronic inflammation-related diseases in various organs [10, 13, 14, 21–23]. Ongoing investigations of the cGAS–STING pathway suggest that agonists or inhibitors associated with this pathway are effective in the treatment of several inflammation-induced diseases.

## The activation of the cGAS–STING pathway and its features

cGAS, a cytoplasmic DNA sensor or receptor that binds directly to DNA, is composed of approximately 520 amino acids [19]. It has a stable and active dimeric structure and its interactions with DNA have been confirmed in X-ray crystallographic studies [26, 27]. Dimer formation is an allosteric process and changes the state of cGAS from inactive to active [28, 29]. Notably, double-stranded DNA (dsDNA) can be sandwiched between these dimers [19].

**Fig. 1:**
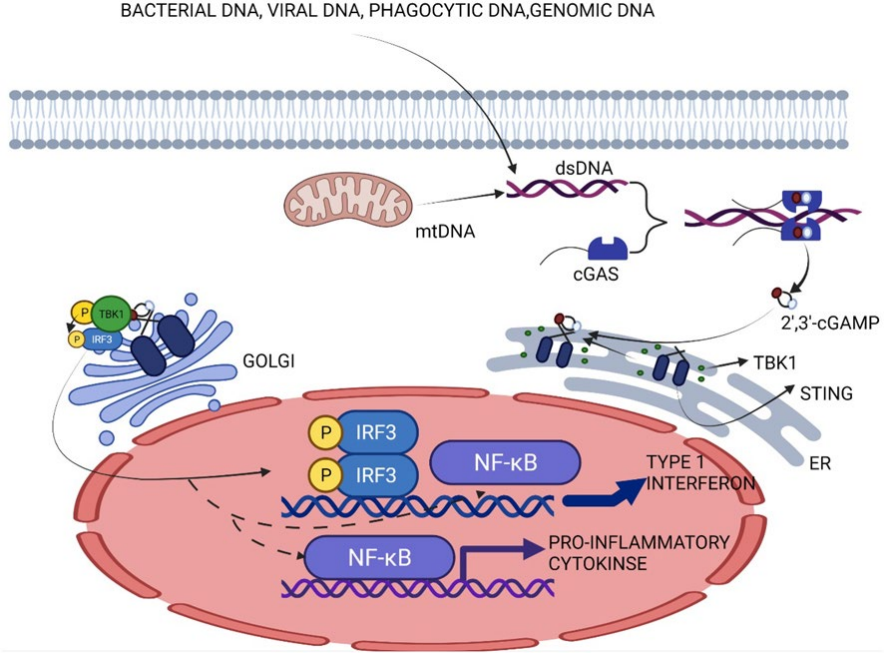
Activation of the cGAS–STING pathway in cells. cGAS is stimulated by foreign double-stranded DNA, mtDNA in the cytoplasm, and phagocytosed DNA. Occupation of the substrates ATP and GTP catalyzes the formation of cGAMP via the induction of cGAS, which subsequently binds to STING located on the ER membrane. The incorporation of cGAMP into STING facilitates the trafficking of STING to the Golgi apparatus. Over the course of translocation, STING recruits and activates TBK1, which subsequently catalyzes the phosphorylation and nuclear translocation of IRF3 and, to a lesser extent, of NF-κB, resulting in the increased synthesis of IFN, along with other inflammatory molecules

When cGAS detects dsDNA from various sources (e.g., viral and bacterial DNA [19], mitochondrial DNA (mtDNA) [30], and micronuclear DNA [31–34], etc.), as a response, it is activated and catalyzes the formation of 2′3′-cGAMP through a series of catalytic reactions and conformational changes (Fig. 1). These reactions are simply divided into two steps: in the first step, cGAS uses adenosine triphosphate (ATP) and guanosine triphosphate (GTP) as precursors and moves ATP to the 2′OH of GTP to generate linear isodinucleotide phosphate pppG (2′–5′) pA, which serves as the base material for the second step [35]; cGAS transfers the GTP to the 3′OH of adenosine phosphate to finally generate cGAMP [36]. The activation of cGAS is not modulated at the transcriptional level, but is modified at the post-translational level, while a number of other mechanisms are involved, including through ubiquitination, acetylation, phosphorylation, and cleavage by cysteinase. These modifications regulate cGAS activity by different mechanisms, e.g. direct modification, removal of the active site, stabilization of the protein, etc. [37–48].

cGAS activates STING and its downstream pathways via cGAMP. It has been shown that inactive STING dimers are present in the ER, and many TBK1 molecules can bind to STING dimers to form STING-TBK1 inactive complexes [49, 50]. Upon binding of cGAMP to the STING-TBK1 complex, the STING dimer undergoes a spatial transition and triggers phosphorylation/activation of the transcription factor IRF3 [49, 50] (Fig. 1). Once IRF3 is activated by the active STING-TBK1 complex, it enters the nucleus and activates downstream reactions [51, 52]. Of note, STING can induce nuclear factor kappa-light chain-activated B-cell enhancer (NF-κB), but in experiments it was shown that the human and mouse STING alleles induced a strong IRF3 response and a relatively weak NF-κB response [53].

Downstream signaling of the cGAS–STING pathway controls interferon (IFN) and NF-κB (a transcription factor that drives expression of pro-inflammatory genes including cytokines and chemokines) expression and promotes the activation of proinflammatory molecules [53–55]. Activated IFN can lead to the upregulation of several hundred IFN-stimulated genes (ISG) [56–58], which in turn promotes the secretion of several pro-inflammatory cytokines. These substances can limit viral replication and induce apoptosis.

Several studies have revealed a promising potential of cGAS use in non-infectious diseases. For instance, in addition to catalyzing the production of second messengers in the nucleus, cGAS can also restrain the restoration of DNA double-strand breaks (DSBs) through homologous recombination without STING or the catalytic activity of cGAS [37, 38]. This is supported by results indicating that the knockdown of *cGAS* inhibits both DNA damage and tumor outgrowth in vitro and in vivo [37, 38]. Preliminary studies have also shown that only adequately long dsDNA can make the cGAS oligomers bind to DNA with high affinity and produce subsequent activity [37]. Based on the above findings, cGAS can be considered as an antitumor factor, while the targeted recruitment of cGAS can be a potential new direction for tumor therapy.

STING can initiate autophagy [59, 60], and it promotes cell survival by maintaining normal autophagic activity after hydrogen peroxide treatment [61]. Despite the fact that STING is implicated in the mediation of autophagy, there is a lack of accurate insight into the mechanism of STING-induced autophagy. Similarly, the cGAS–STING pathway participates in the regulation of cellular senescence [33, 62], an irrecoverable cell cycle stalling that occurs in response to severe cellular stress. In addition to inhibiting auto-proliferation, senescent cells produce inflammatory cytokines, growth factors, and proteases. This phenotype is known as the senescence-associated secretory phenotype (SASP); it strengthens senescence in an autocrine mechanism and mediates growth inhibition via a paracrine mechanism. In addition, chemokines of SASP enable the activation and recruitment of immune cells to destroy abnormal cells with damaged DNA [63, 64]. In the absence of cGAS as well as STING, both SASP induction and immune cell infiltration are defective [62].

Finally, the cGAS–STING pathway can cell type-specific induce apoptosis [65], STING can induce the expression of many pro-apoptotic molecules [66]. In the cGAS–STING pathway, activated IRF3 can combine with BCL-2 related X protein (BAX) to form a complex that directly induces apoptosis [67]. Furthermore, STING is also involved in the induction of the lytic cell death program [68]. It can activate mixed-lineage kinase domain-like pseudokinase (MLKL) [69], an enzyme involved in the induction of necrosis. p53 upregulated modulator of apoptosis (PUMA) [70] is activated in an MLKL-dependent manner, which exacerbates mtDNA leakage in mitochondria and thus leads to programmed cell death.

## Major identified diseases associated with the cGAS–STING pathway

### The cGAS–STING pathway and infectious diseases

The cGAS–STING pathway is primarily activated by dsDNA that enters the cytoplasm abnormally; therefore, it is tempting to emphasize the connection between the cGAS–STING pathway and viral infections, such as those caused by classical herpes simplex virus type 1 [71]. Considerable research results are available, but as they have little relevance to the topics described in this review, only some reviews are provided for reference only [8, 9]. Notably the study found that mouse hepatocytes lack STING, but the application of selective expression of STING in mouse hepatocytes elevated the IFN response to DNA stimulation and enhanced the control of hepatitis B virus (HBV) [72].

## The cGAS–STING pathway and cancer

Given how sensitive the cGAS–STING pathway is to abnormal intracellular DNA, researchers are interested in investigating the effects of this pathway on cancer. As research has progressed, scientists have obtained fruitful results regarding the associations between the cGAS–STING pathway and tumors. Given that it is not the subject of this review, here, the authors provide some reviews on the relationship between the cGAS–STING pathway and tumors [10, 11]. Among the studies on the relationship between the cGAS–STING pathway and tumors, it was surprising that researchers found that the cGAS–STING pathway might be relevant to some tumor metastasis [73]. The discovery reflects the complexity of the cGAS–STING pathway in regulating disease processes and alerts researchers to the necessity of more rational application of cGAS–STING pathway-related drugs for treatment.

## The cGAS–STING pathway and autoimmune diseases

Autoimmune diseases are conditions that arise when the body abnormally attacks its own normal cells. At least 80 such disorders have been identified, and they can occur in almost any part of the body. Common symptoms include intermittent fever and fatigue. Some autoimmune diseases have been linked to mutations in genes that regulate the cGAS–STING pathway. For example, patients with vascular and pulmonary syndrome (VAPS) [74], a systemic inflammatory disease, have mutations in STING exons. Other genetically induced autoimmune diseases include STING-associated vasculopathy with onset in infancy (SAVI) [74–77]; Aicardi-Goutieres syndrome [78, 79], systemic lupus erythematosus (SLE) [80, 81], familial cerebellar lupus erythematosus [82, 83], and polyarthritis induced by deoxyribonuclease II (DNaseII) gene defects [84–86]. Table 1 lists these diseases and the genes involved.

**Table 1:** cGAS–STING-related autoimmune diseases

STING-related gene mutations	TREX1 mutation	DNase II mutation
VAPS	AGS	Polyarthritis
SAVI	SLE	
	Familial cerebellar lupus erythematosus	
	Retina with leukodystrophy Vascular disease	

*VAPS* vascular and pulmonary syndrome, *AGS* Aicardi-Goutieres syndrome, *DNaseII* deoxyribonuclease II, *SLE* systemic lupus erythematosus, *SAVI* STING-associated vasculopathy with onset in infancy, *TREX1* three prime repair exonuclease 1

Because early knowledge of the cGAS–STING pathway was not comprehensive initially, the studies conducted mostly focused on viral diseases, cancer, and autoimmune diseases. However, as research on the cGAS–STING pathway progressed, researchers tended to link the cGAS–STING pathway to chronic diseases. Although the cGAS–STING pathway is still most intensively studied in relation to viruses, targeting the cGAS–STING pathway in exploring the pathogenesis of chronic diseases is an attractive research direction.

## Novel physiological and pathological roles of the cGAS–STING pathway in specific organs

With the knowledge of mechanisms related to the cGAS–STING pathway, researchers have gained a better understanding of diseases caused by low-grade inflammation, and as a result, some investigators have initiated investigations one the cGAS–STING pathway in different organs. Some of the research advances in these areas are presented below.

## Heart

Heart disease is one of the most common causes of death worldwide, and the etiology of many common heart diseases is still not fully understood, but inflammation, fibrosis, and oxidative stress are known to contribute to heart-related diseases. Given the impressive performance of the cGAS–STING pathway in inflammation, many research groups have directed their work to the function that the cGAS–STING pathway performs in different cardiac diseases.

First identified was that the cGAS–STING pathway was more expressed in the hearts of SLE patients compared to normal subjects [87]. In a subsequent study, a research group found that STING-IRF3 was activated and induced an inflammatory response in endothelial cells treated with palmitic acid (PA) [88]. In addition to the cGAS–STING pathway being shown to be activated in vitro, the cGAS–STING pathway was shown to exacerbate the pro-inflammatory response in a mouse model of myocardial infarction [89]. In contrast, myocardial function and survival were improved in myocardial infarct mice when cGAS and STING were absent [89]. Not only limited to myocardial infarction, high expression of the cGAS–STING pathway in different causes of cardiac dysfunction has been shown to be involved in the development of cardiac dysfunction, and cardiac function is significantly protected when the cGAS–STING pathway can be inhibited [90–92].

Not only limited to cardiac energy metabolism and the coronary system, the cGAS–STING pathway also contributes to the abnormal cardiac function caused by structural abnormalities of the heart. STING was found to be more highly expressed in cases of dilated cardiomyopathy (DCM) and hypertrophic cardiomyopathy (HCM) as well, and cardiac hypertrophy and fibrosis were significantly attenuated in DCM and HCM when STING was inhibited. This result suggests that STING serves as a pivotal link in pathological myocardial hypertrophy [93]. The effect of the cGAS–STING pathway on myocardial structural changes is not limited to cardiomyopathy but is also seen in transverse aortic systolic surgery (e.g., mouse models) [23]. In contrast, when cGAS expression is suppressed, fibrosis, myocardial hypertrophy and apoptosis are reduced, and cardiac function is significantly improved [23].

Researchers have found some clues as to the mechanism by which the cGAS–STING pathway functions in the heart, but further studies are still needed to clarify the mechanism. For instance, leakage of mtDNA into cytoplasm is thought to be a possible factor in the activation of the cGAS–STING pathway in the heart. Studies have shown a clear association between mtDNA and cardiac dysfunction [94]. Whereas in earlier studies mtDNA stress was found to trigger a significant antiviral innate immune response, mtDNA leakage in the cytoplasm could enhance the cGAS–STING pathway and thus the inflammatory response [30]. In studies on endothelial cells, researchers found that PA activates the cGAS–STING pathway by causing mitochondrial damage and mtDNA release into the cytoplasm [88]. This study did not further confirm the link between mtDNA and cGAS–STING pathway in animal models, but the results of this experiment also provide ideas for the mechanism of cGAS–STING pathway activation in heart disease. ER stress has also been suggested as a crucial mechanism for the role of the cGAS–STING pathway in cardiac diseases. Cardiac hypertrophy and fibrosis were significantly attenuated when STING was inhibited in mice with cardiac hypertrophy [93], whereas there was a significant increase in STING expression when ER stress activators were applied in in vitro experiments [93]. These findings suggest that alterations in STING expression correlate significantly with ER stress and may promote the progression of cardiac hypertrophy when STING expression is increased. Therefore, it is necessary to further investigate the link between the cGAS–STING pathway and ER stress and its mechanisms in appropriate models.

In addition to mtDNA leakage and ER stress, researchers found in mouse models of myocardial infarction that the expression of some key inflammatory substances (e.g., inducible nitric oxide synthase; iNOS) was reduced in the absence of cGAS and STING and could contribute to the conversion of macrophages to a repair phenotype, which improved the survival of myocardial infarction mice [25, 95]. These experimental results suggest that the cGAS–STING pathway may be involved in cardiac disease by altering macrophage polarity.

In conclusion, the cGAS–STING pathway exhibits a function in cardiac disease as exacerbating cardiac disease, and the mechanisms by which the cGAS–STING pathway is involved in cardiac disease are complex, requiring researchers to conduct additional studies to elucidate the role played by the cGAS–STING pathway in the progression of cardiac disease. This may also provide important assistance for the precise use of inhibitors related to the cGAS–STING pathway.

## Lung

Many lung diseases are thought to be correlated with inflammation and autoimmune hyperactivation, and the cGAS–STING pathway has been introduced into the exploration of the mechanisms of these lung diseases because of its manifestation in diseases associated with inflammation. These diseases include chronic obstructive pulmonary disease (COPD), pulmonary fibrosis and asthma.

COPD is an aggressive, chronic inflammatory disease related to the impairment of lung function, which is characterized by persistent long-term inflammation, typically as a result of excessive production of reactive oxygen species, leading to oxidative stress and DNA damage. There have been some developments regarding the association between COPD and the cGAS–STING pathway. In one study, researchers found that cigarette smoke caused respiratory barrier damage in mouse model, which resulted in the release of DNA into the cytoplasm. This activated the cGAS–STING pathway, which in turn triggered type I IFN-dependent lung inflammation. These effects were significantly attenuated in mice lacking STING [97].

However, another group of researchers found that STING expression was decreased in mice exposed to cigarette smoke or smoke extracts. The experiments were conducted by exposing mice to cigarette smoke to develop a COPD model and then infecting the mice with recombinant adenoviral vectors (rAdVs) to develop a model of acutely exacerbated COPD (AECOPD). In the experiment, STING expression was suppressed in mice exposed to cigarette smoke + infected with rAdVs compared to rAdVs-infected mice. In addition when STING was knocked out, the lung inflammatory response and fibrosis were increased in cigarette smoke + rAdVs infected treated mice [98]. This result suggests that STING can protect lung function in rAdVs-infected mice under cigarette smoke exposure. Not limited to animal models, another study confirmed that ISG expression was detectable in both stable COPD patients and healthy subjects, while ISG expression was reduced in patients with AECOPD [96]. Based on the above experimental results, we conjecture that, given the role of STING in both antiviral and inhibition of pulmonary fibrosis, STING is used to fight viral infection when the body is infected with a virus, and the corresponding inhibition of inflammation and fibrosis may be compromised, which may lead to an exacerbation of disease progression in AECOPD. The mechanism for the different results in the two groups of mice exposed to cigarette smoke is also unclear and may be related to the different duration of smoke treatment in the two groups. What is confirmed is that STING can have a relatively important contribution to disease progression in COPD, and that it may not play the same role in acute and chronic COPD.

In addition to its involvement in COPD, cGAS–STING also plays a significant role in pulmonary fibrosis. Silica particles cause lung inflammation and fibrosis, and in a 2018 study, STING was experimentally confirmed as a critical component in silica-induced lung disease. Patients with silicosis had significantly higher plasma levels of dsDNA than normal subjects, and the cGAS–STING pathway was activated in the lungs of these patients and mice exposed to silica particles [99]. In animal experiments, silica particles triggered lung cell death and the release of dsDNA into the bronchoalveolar space leading to STING expression, which may be a possible mechanism by which STING is activated [99]. Idiopathic pulmonary fibrosis is a fatal interstitial lung disease, a form of pulmonary fibrosis characterized by the irreversible destruction of lung structures, which in turn leads to respiratory failure and death. A group of investigators found that ER stress was triggered, and STING expression was decreased in patients diagnosed as having acute exacerbations of idiopathic fibrosis and in pulmonary fibrosis mouse models. When treated, STING levels significantly increased in patients with pulmonary fibrosis [100]. Reportedly, STING deficiency leads to increased collagen deposition in the lung and increased pulmonary fibrosis, reflecting the protective role of STING in idiopathic pulmonary fibrosis [101]. The different expression levels of STING in silica-induced and idiopathic pulmonary fibrosis show that there may be different cGAS–STING regulatory mechanisms for different etiological conditions. This requires further investigation of the various regulatory mechanisms involved.

Asthma is a chronic inflammatory lung disease involving multiple cells (e.g., eosinophils, mast cells, T lymphocytes, neutrophils, and airway epithelial cells) and cellular components. It is characterized by airway hyperresponsiveness and reversible impaired ventilation. Common exacerbation triggers include viral infections and dust mites. In an experiment conducted in an asthma mouse model, the accumulation of cytoplasmic dsDNA in airway endothelial cells increased in mice in an allergic state. Furthermore, the absence of cGAS significantly attenuated allergic airway inflammation caused by chicken ovalbumin or house dust mites (HDM) [102]. Therefore, the cGAS–STING pathway probably contributes to the immune response at the onset of asthma; However, whether cGAS itself is involved in this immune response, or whether cGAS activates the immune response through the cGAS–STING pathway, further studies are necessary to confirm. Other groups have also examined gene expression in patients with mild and severe asthma and confirmed that ISG levels were increased in these patients and that increased airway ISG expression was associated with decreased lung function. More importantly, by detecting ER stress-related proteins, the experiments show that ISG was associated with ER stress markers through the detection of ER stress-related proteins, implying that there may be a potentially link between ISG-induced inflammation and ER stress [103], which requires further de-confirmation.

In conclusion, further confirmation of the function of the cGAS–STING pathway during the development of various lung conditions is required. It is also crucial to further elucidate the effect of cGAS–STING pathway-mediated cytokine secretion in patients with pulmonary conditions.

## Liver

Numerous studies have confirmed the link between the liver and the cGAS–STING pathway. In addition to HBV and liver cancer, the cGAS–STING pathway has been shown to be activated in other kinds of liver diseases (e.g., non-alcoholic fatty liver disease (NAFLD), alcohol liver disease (ALD), liver ischemia–reperfusion, etc.).

In the previous section, we have described that STING-IRF3 can be activated in PA-induced endothelial cells, and this experiment also reported that in high-fat diet (HFD) mice, STING-IRF3 is also activated and that both HFD-induced inflammation and obesity are suppressed to some extent when STING is absent [88]. And in different models of nonalcoholic steatohepatitis, STING deficiency attenuated hepatic steatosis, inflammation and fibrosis [104]. In addition to animal models, it has also been demonstrated that increased STING expression has been observed in Kupfer cells from nonalcoholic steatohepatitis patients [105]. A report of a trial on 98 participants confirmed that patients with NAFLD had higher levels of STING in liver tissue, along with higher levels of inflammation and fibrosis, compared to the control group [106].

ALD is a common indication for liver transplantation. In a 2016 study on liver fibrosis in mice, ER stress promoted IRF3 activation and resulted in hepatocyte apoptosis and injury. Conversely, both fibrosis and apoptosis were inhibited in mice lacking IRF3 expression due to knocking out STING or IRF3 [107]. This confirmed the role of STING in promoting the progression of liver disease to fibrosis or hepatocyte death in mice. In subsequent studies on ALD, investigators established that the cGAS–STING pathway gets occupied and leads to IRF3 interaction with proapoptotic BAX, which resulted in hepatocyte apoptosis, thus setting the stage for inflammation and injury [108]. Moreover, liver damage was reduced when the cGAS–STING pathway was inhibited [109]. Alcohol-induced cytoplasmic leakage of mtDNA amplifies the cGAS–STING pathway, which has been shown to amplify liver injury through hepatic gap junctions in ALD [109]. In view of the above experimental results, although the mechanism of the effect of the cGAS–STING pathway on fatty liver (including NAFLD and ALD) is not fully elucidated, it is possible to suggest that the cGAS–STING pathway could be a potential therapeutic target for the treatment of fatty liver.

With the development of liver transplantation, liver ischemia–reperfusion has received increasing attention. Of note, individual proteins of the cGAS–STING pathway are implicated in this process. The researchers found that cGAS itself can have a protective effect on the liver. This protective property is independent of STING, a process that protects hepatocytes from apoptosis after oxidative stress [110]. Another study in 2020 with a mouse model of ischemia–reperfusion confirmed that in macrophages, STING can promote the activation of NLRP3 which can exacerbate liver ischemia–reperfusion injury [111]. Taken together, the cGAS–STING pathway is of great influence on the occurrence of diseases in the liver. These studies on cGAS–STING in the liver provide new insights into our understanding of the largest visceral organ.

## Kidneys

Acute or chronic kidney lesions may be associated with the cGAS–STING pathway. As of 2017, a study conducted on SLE by a group of researchers confirmed that tumor necrosis factor (TNF)-like weak inducer of apoptosis could activate type I IFN in lupus nephritis (LN) causing kidney injury. This demonstrated that type I IFN has a role in kidney injury caused by autoimmune diseases [112]. In a subsequent study, Japanese scholars found that in cisplatin-induced acute kidney injury mouse model, the cGAS–STING pathway got triggered by mtDNA and elicited inflammatory injury [113]. Conversely, the suppression of inflammation was observed in *STING*-knockout mice, thus confirming the usefulness of the cGAS–STING pathway in the pathogenesis of acute kidney disease [113].

In a 2019 study, researchers also affirmed the contribution of the cGAS–STING pathway to the development of chronic kidney disease. Researchers initially observed that chronic kidney disease recruits activation of the cGAS–STING pathway, and that this activation is associated with abnormalities in mitochondrial metabolism [114]. Subsequently, they also observed that fibrosis and renal failure were suppressed in STING knockout mice [114]. These experiments confirm the critical role of the cGAS–STING pathway in renal inflammation due to abnormal mtDNA accumulation, but do not provide sufficient evidence to define whether the mobilization of the cGAS–STING pathway is dependent on the amount of mtDNA leakage. Another study on the development of LN-related end-stage renal disease in African-Americans also confirmed the essential role of the cGAS–STING pathway in renal disease [115]. This pathway involved IFNs plus nucleosome-associated dsDNA (nsDNA) fragments and triggered the expression of IFN-β. Furthermore, the inhibition of STING suppressed the overexpression of human nsDNA.

In summary, the abovementioned findings demonstrate the vital role the cGAS–STING pathway play in acute to chronic kidney lesions by increasing the expression of individual cGAS–STING pathway-associated proteins upon the occurrence as well as suppression of damage in models after cGAS–STING pathway-associated proteins are knocked out. This indicates that precise targets of the cGAS–STING pathway can be potentially useful in kidney-related diseases.

## Other organs

There is now some evidence that the cGAS–STING pathway is activated in diseases in organs other than those discussed above; for example, in the nervous system, the cGAS–STING pathway is activated in traumatic brain injury, ischemic stroke, and Huntington's disease [116–118]. Moreover, the activation of the cGAS–STING pathway is also correlated with diseases such as age-related macular degeneration, aortic degeneration, small angiogenesis, acute pancreatitis, and colitis [119–123].

**Fig. 2:**
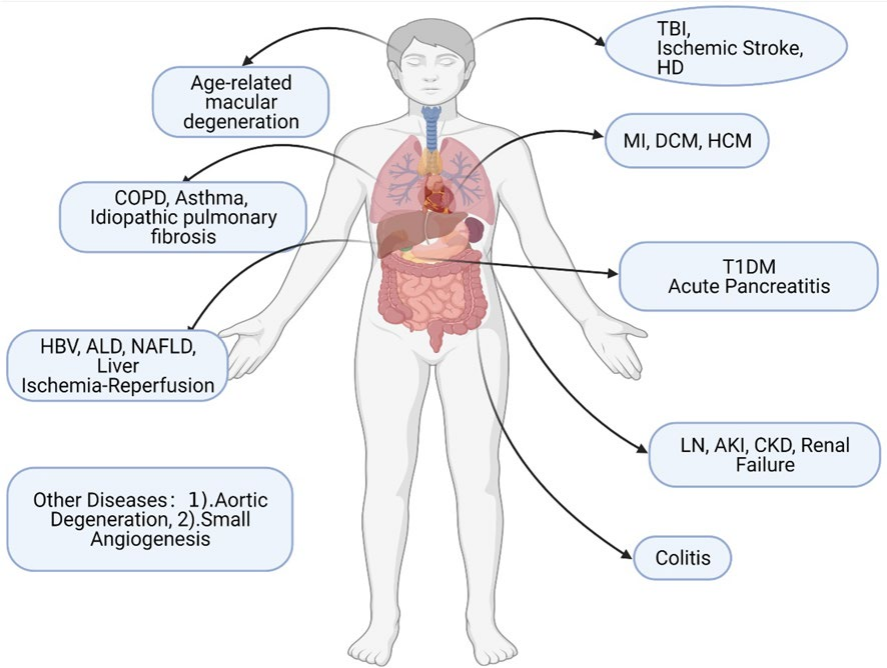
Novel physiological and pathological roles of the cGAS–STING pathway in specific organs (not including cancer and some infectious diseases). With the current understanding of the mechanisms associated with the cGAS–STING pathway, researchers have gained more insight into some of the diseases caused by inflammation and have studied these diseases. The figure shows some of the identified diseases associated with the cGAS–STING pathway. *AKI* acute kidney injury, *ALD* alcoholic liver disease, *CKD* chronic kidney disease, *COPD* chronic obstructive pulmonary disease, *DCM* dilated cardiomyopathy, *HBV* hepatitis B virus, *HCM* hypertrophic cardiomyopathy, *HD* Huntington’s disease, *LN* lupus nephritis, *T1DM* type 1 diabetes mellitus, *MI* myocardial infarction, *NAFLD* nonalcoholic fatty liver disease, *TBI* traumatic brain injury

Overall, the cGAS–STING pathway exerts a complex impact on the progression of these diseases, not only via inflammatory damage promotion, but also, in some respects, through a protective function, making it difficult to identify the pathway that is more important in the development of the diseases. We can speculate that the inhibition of cGAS–STING in multiple organs (Fig. 2) may reduce the severity of some diseases; however, it may also significantly downregulate innate immune function, leading to an increased risk of viral infection and tumor invasion. Therefore, in addition to investigating the mechanisms of action of cGAS–STING, it is important to study how the body precisely regulates the pathways involving these proteins.

## Role of the cGAS–STING pathway in diabetes

Diabetes imposes a significant economic burden on individuals and the society. This is attributed to the increased prevalence of diabetes and the increased cost per patient with diabetes [124]. Although there have been tremendous advances in diabetes research in recent years, including extensive research on the mechanisms and treatment of diabetes, a high prevalence of diabetes and related complications remains. Therefore, there is an urgent need to develop more effective therapies.

## Diabetes and inflammatory responses

diabetes is a metabolic disease affecting multiple organs and is mainly caused by absolute or relative insulin deficiency which mainly manifests as reduced glucose tolerance and hyperglycemia. Empirical evidence indicates that inflammatory pathways can contribute to obesity, insulin resistance (IR), and its subsequent diabetes-related metabolic disorders and complications. Therefore, the targeted administration of anti-inflammatory therapy may be helpful in the prevention of diabetes, and the application of tests to detect relevant inflammatory indicators may help understand the prognosis of diabetes and metabolic disorders.

Type 1 diabetes mellitus (T1DM) is an autoimmune disease characterized by the destruction of pancreatic islet β-cells leading to inadequate insulin synthesis. The involvement of immune cells in the progression of T1DM has been demonstrated in several animal models. As in the case of the T1DM model of transmissible T cell transfer, it has been shown that T cells are capable of destructive peri-islet inflammatory infiltration and diabetes induction [125, 126]. In addition, macrophages are key mediators of islet inflammation owing to their ability to secrete cytokines such as IL-1β and TNF-α. These two cytokines can act synergistically with IFN-γ, leading to the uptake of iNOS and the consequent generation of nitric oxide (NO) [127]. The mechanisms mentioned above may lead to pancreatic β-cell death in multiple ways; therefore, the control and regulation of local inflammatory cytokine production may be a key factor in determining the outcome of T1DM progression.

Type 2 diabetes mellitus (T2DM), in contrast, is characterized by a relative scarcity of insulin, which manifests as IR, a state in which essential organs, such as the liver and muscles, are less sensitive to insulin. Fat accumulation in the liver and muscle tissues is the antecedent of IR and precedes the onset of T2DM. Moreover, fat accumulation exacerbates IR and causes dysfunction of pancreatic β-cells at the late stage of T2DM [128]. It is now well-established that obesity and its associated MetS are positively associated with the induction of inflammatory responses (mediated by NF-κB) which contribute to the development of IR and T2DM [129]. Adipokines (such as leptin and adiponectin) stimulate other inflammatory responses in individuals with obesity, and macrophages and immune cells can then infiltrate adipose tissue [130–133]. A combination of these factors results in low levels of sustained inflammatory responses in the body, eventually leading to diseases related to organ damage and dysfunction.

Therefore, it is imperative to focus on diabetes prevention and treatment research. Notably, inflammatory pathways are under consideration as treatment targets in diabetes and its complications.

## Protein kinase B (Akt) and diabetes

**Fig. 3:**
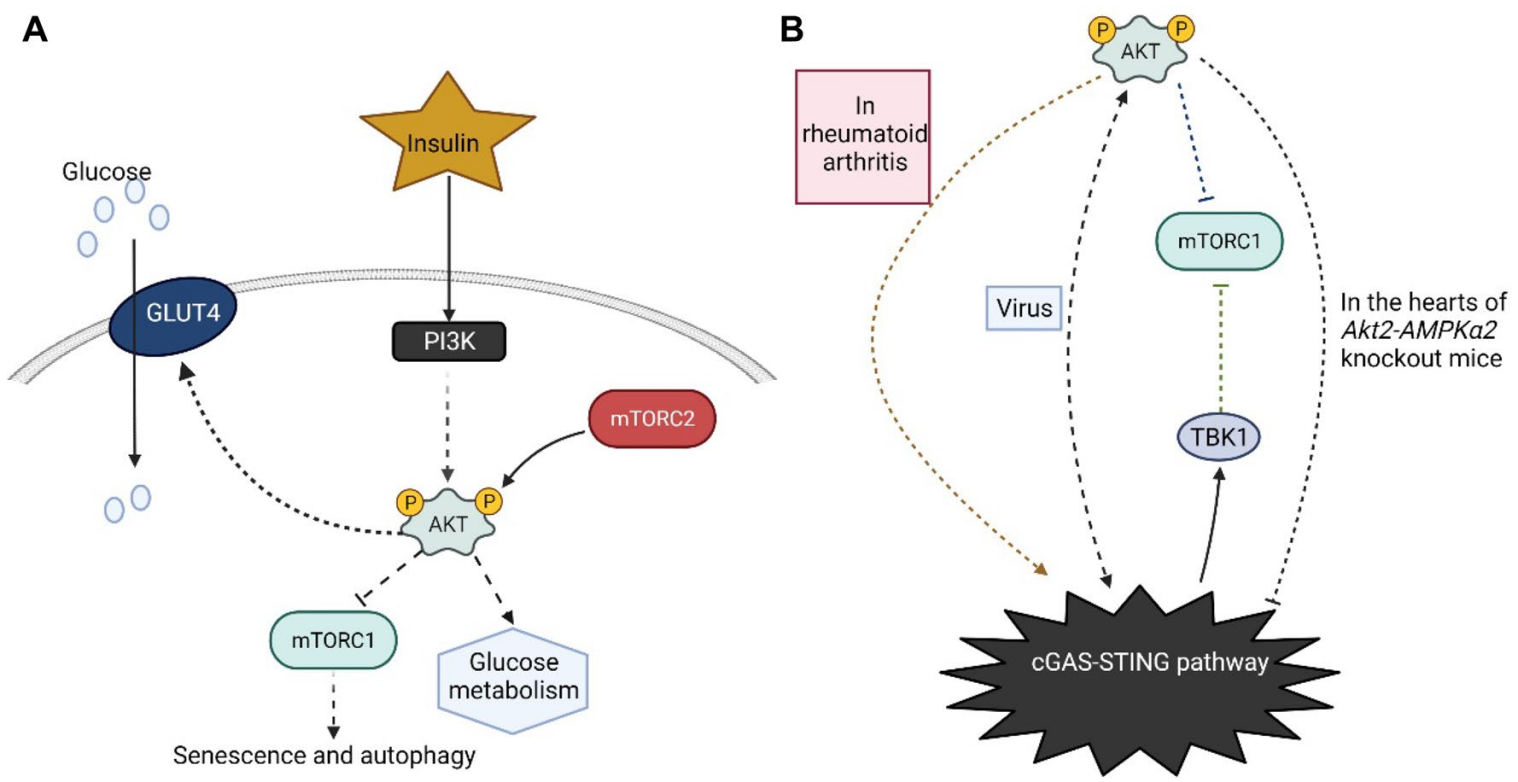
The cGAS–STING pathway is linked to diabetes through Akt. **A** Insulin-mediated activation of Akt is essential for glucose handling and metabolism. Insulin activates PI3K/Akt. Akt is activated and phosphorylates many of its substrates, which regulate glycogen synthesis, gluconeogenesis, and glucose uptake. Akt can regulate cellular uptake of glucose by stimulating GLUT4 from cytoplasm to cell membrane. mTOR is a serine/threonine kinase that responds to a variety of environmental stimuli, including nutrient, oxygen, and energy levels, and forms two functionally distinct complexes, mTORC1 and mTORC2. mTORC1 can act as a nutrient/energy/redox sensor, controlling protein synthesis and growth factors. Prolonged activation of mTOR1 by glucose/fatty acids can further promote cellular damage, thereby exacerbating diabetes-associated cell and tissue damage while mTORC2 may also activate Akt function. **B** The link between the cGAS–STING pathway and Akt was shown earlier in rheumatoid arthritis, where Akt expression was significantly decreased when cGAS expression was suppressed [149]. However, in virus infection conditions, the relationship between the cGAS–STING pathway and Akt exhibits differently. Akt activation reported in the earlier experiments inhibited the cGAS–STING pathway [41], but in later experiments, activated Akt positively regulated the cGAS–STING-mediated antiviral action [150, 151]. Reportedly TBK1 is able to inhibit mTORC1 activity in mice [153]. Given that the role of mTORC1 can be regulated by Akt and TBK1 (a downstream link of the cGAS–STING pathway), mTORC1 may be a key link connecting the cGAS–STING pathway to Akt. Limited information indicates that in HFD-treated Akt2-AMPKα2 double-knockout mouse hearts, Akt2-AMPKα2 double-knockout activated the cGAS–STING pathway, as the first evidence for the link between Akt and cGAS–STING pathway in metabolic disease. To date, there remains no study on whether knockdown of Akt alone activated the cGAS–STING pathway. Therefore, whether the cGAS–STING pathway functions in the pathogenesis of diabetes, whether the cGAS–STING pathway plays a role in the pathogenesis of diabetes, and what function does Akt play in these diseases all need to be further investigated. *AMPK* AMP-activated protein kinase, *Akt* protein kinase B PKB, *Akt2* Akt serine/threonine kinase 2, *GLUT4* glucose transporter type 4, *mTORC1* mammalian target of rapamycin complex 1, *mTORC2* mammalian target of rapamycin complex 2, *PI3K* phosphatidylinositol 3 kinase. The solid lines and dash lines indicate direct and indirect action, respectively

Insulin is the most important substance in metabolism regulation because it regulates the production of glucose, lipids, and proteins mainly through the phosphatidylinositol 3-kinase (PI3-K) pathway [134]. Substrate proteins of the insulin receptor are phosphorylated, resulting in binding and activation of PI3-K, which activates downstream kinases, like phosphatidylinositol-dependent kinases [134], and consequently initiates Akt expression (Fig. 3). Akt is an important protein involved in insulin signaling, metabolism, cell growth, and the cell cycle. It mediates glucose transportation via glucose transporter-4 (GLUT-4) molecules into cells that metabolize glucose in an insulin-dependent manner [134]. Akt1 inhibits apoptosis and improves cell survival [135]. Akt2 is involved in the insulin signaling pathway [136] as evidenced by the development of IR and T2DM-like symptoms in human with an Akt2 deficiency [137]. Although there are reviews that describe the role of Akt in diabetes [138], additional studies now suggest that the regulation of Akt in diabetes may not be as simple a process as considered previously. mTOR is a serine/threonine kinase that responds to various environmental stimuli, including nutrients, oxygen, and energy levels and forms two functionally distinct complexes, mTORC1 and mTORC2. The mTOR pathway integrates inputs from upstream pathways, including insulin, growth factors and amino acids, while mTOR also senses cellular nutrient, oxygen and energy levels. mTORC1 can act as a nutrient/energy/redox sensor that controls protein synthesis and growth factors that can trigger the activation of mTORC2, whereas mTORC2 can activate Akt1 which can activate mTORC1 indirectly (Fig. 3) [139]. The chronic activation of mTOR by glucose/fatty acids can further promote the apoptosis of pancreatic β-cells, thereby exacerbating diabetes progression. From the above brief description, we can find that the function of the Akt protein family is very complex and may contain multiple mechanisms; and shows crosstalk with many other signaling pathways. These complex mechanisms are focused in diabetes as shown by the remarkable contribution that the Akt protein family can make in suppressing IR and increasing insulin sensitivity.

The complex functions exhibited by the Akt protein family undoubtedly endow the cells with powerful capabilities. However, this complex crosstalk also makes it difficult to predict the possible network-wide effects of diabetes treatment targeting pathway nodes. Given the diverse roles of the Akt protein family in diabetes, we must continue to investigate and uncover all possible feedback mechanisms and pathway interactions. It is equally essential to understand the initiating factors of diabetes and how these factors impact these mechanisms and interactions as well as how the various Akt-interacting signaling pathways support and compensate for each other.

## Adiponectin and disulfide bond A oxidoreductase-like protein (DsbA-L)

Adipocytes secrete various adipocytokines; adiponectin, in particular, is a protein that plays a key role in sugar and lipid metabolism. It has 244 amino acids and has been identified as a crucial factor for the regulation of energy homeostasis through the employment of peroxisome proliferator-activated receptors (PPARs) [140] and AMP-activated protein kinase (AMPK) [141]. Owing to its key part in metabolism, adiponectin is considered an important therapeutic target in diabetes and MetS.

PPAR promotes the expression of adiponectin genes in diabetic mice and improves insulin sensitivity by increasing fatty acid utilization and reducing triglyceride levels in the liver and skeletal muscles [140, 142]. Additional evidence of decreased hepatic insulin sensitivity and poor response to agonists of PPARs in adiponectin-deficient mice suggests that adiponectin can slow the progression of diabetes by directly affecting insulin sensitivity [143]. In addition, the AMPK pathway can be activated by adiponectin to inhibit acetyl coenzyme A carboxylase, leading to increased fat oxidation and glucose transporter expression [144]. AMPK can promote glucose uptake by increasing GLUT-4 transport to the plasma membrane [145], whereas in adipose tissues, AMPK activation decreases PPAR expression and reduces adipogenesis by increasing lipolysis. It also suppresses the discharge of TNF-α and IL-6 in the adipose cells as well as increases the secretion of adiponectin [146]. In conclusion, adiponectin can mediate metabolic pathways in the body, and increasing its expression can reduce the risk of diabetes and MetS.

**Fig.4:**
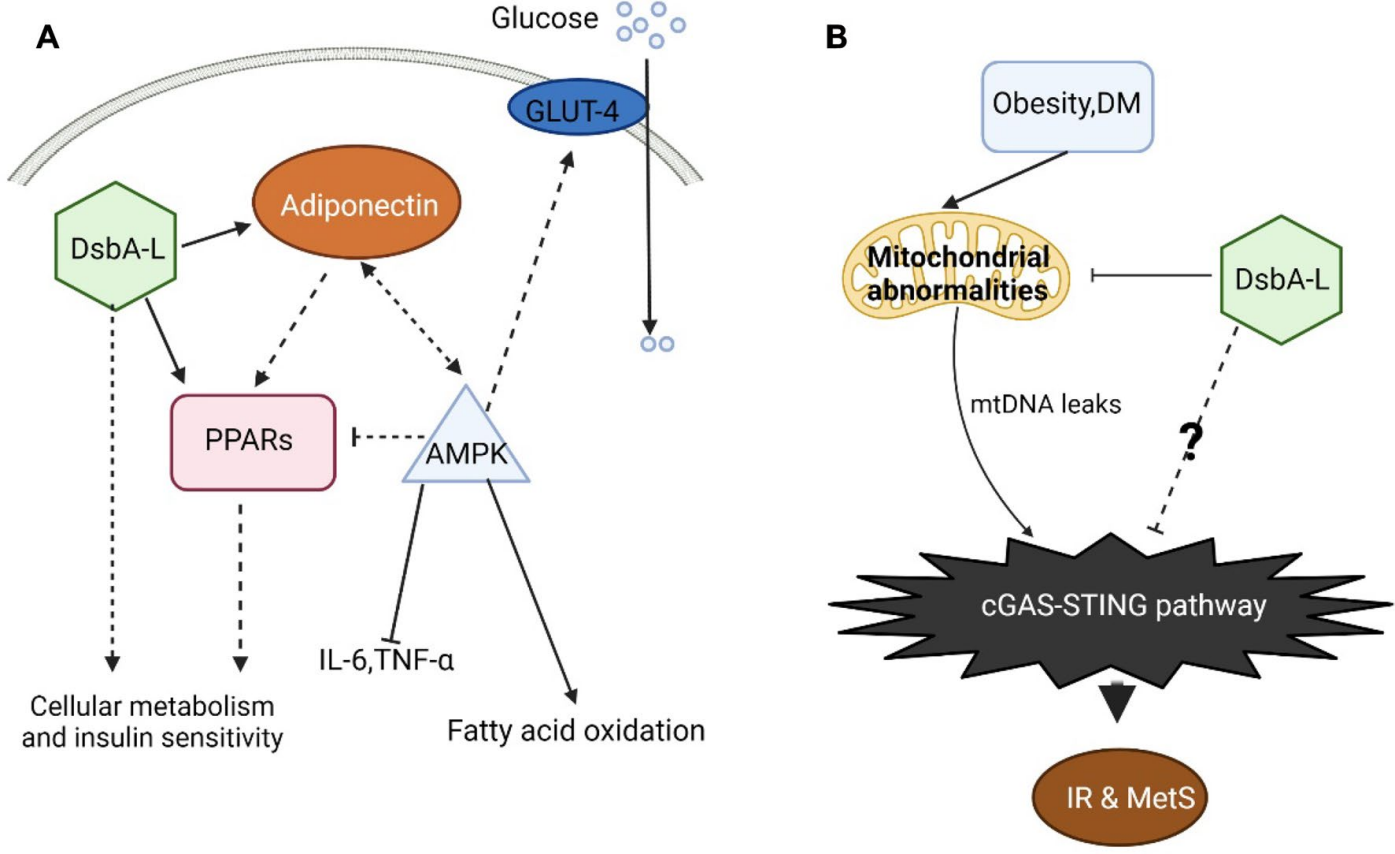
The cGAS–STING pathway is associated with diabetes through DsbA-L. **A** Adiponectin promotes the expression of PPAR-regulated genes and improves insulin sensitivity in diabetic mice [140, 142]. Adiponectin activated the AMPK pathway increases fat oxidation and glucose transporter localization to the membrane [144, 145]. AMPK activation inhibits PPAR expression and TNF-α and IL-6 emission in adipose tissue [146]. In addition to directly promoting adiponectin multimerization, DsbA-L also facilitates energy homeostasis in mice, promoting energy expenditure and reducing obesity, and this regulation is independent of adiponectin. **B** Emerging evidence indicates that DsbA-L directly participates in the maintenance of normal mitochondrial homeostasis and inhibits the cGAS–STING pathway [14]. Obesity can promote mitochondrial damage and leak mtDNA by inhibiting DsbA-L. The leaked mtDNA, in turn, activates the cGAS–STING pathway, which in turn causes IR and metabolic syndrome. Conversely, increased DsbA-L inhibits mtDNA leakage, which in turn inhibits the cGAS–STING pathway. In the same metabolic syndrome as diabetes, we believe that this is likely to be the case as well, but experiments are still needed to confirm this. In addition, whether DsbA-L inhibits the cGAS–STING pathway exclusively by suppressing mtDNA leakage still needs to be further explored. *DsbA-L* disulfide bond A oxidoreductase-like protein, *IR* insulin resistance, *MetS* metabolic syndrome, *mtDNA* mitochondrial DNA, *PPARs* peroxisome proliferator-activated receptors. The solid lines and dash lines indicate the direct and indirect action, respectively

DsbA-L is a molecular chaperone involved in adiponectin multimerization and plays an important protective role in MetS [147, 148]. The adipose-specific overexpression of DsbA-L protects mice from HFD-induced adiponectin downregulation and reduces IR and hepatic steatosis. In addition to protecting mice from the adverse effects of obesity and IR by promoting adiponectin multimerization, DsbA-L appears to protect high-fat-fed mice through a adiponectin-independent pathway. In a study, mice with adiponectin knockout were divided into two groups, one overexpressing DsbA-L and the other serving as a control. No significant differences were observed in the body weights and insulin sensitivities of the same normal diet-fed mice. However, when both groups were fed an HFD, mice with DsbA-L overexpression and adiponectin knockout showed lower body weights and adipocyte volumes than mice with only adiponectin knockout [147]. In addition, the downregulation of DsbA-L expression reduces PPARγ agonist function, suggesting that DsbA-L not only increases insulin sensitivity, but also has a prominent function in the prevention of diabetes (Fig. 4) [148].

The protective function of adiponectin and DsbA-L in metabolism implies the essential use of adiponectin, DsbA-L, etc. as therapeutic targets when treating metabolism-related diseases such as obesity and diabetes. Although there are few studies on the relationship between DsbA-L and diabetes, it is necessary to investigate the molecular pathways through which DsbA-L inhibits the development of diabetes under the complex crosstalk of the whole network.

## The cGAS–STING pathway is linked to diabetes through Akt

There is no direct evidence on whether the cGAS–STING pathway affects diabetes. The available evidence is based on animal experiments in non-obese diabetic (NOD) mice. Non-treated female NOD mice showed signs of T1DM from 16 weeks of age and the incidence of T1DM increased to approximately 90% at 35 weeks of age [24]; however, treatment of the female prediabetic NOD mice with DNA nanoparticles (DNPs) (activators of STING) at 4–8 weeks delayed the progression of T1DM, owing to which all mice remained disease-free until 27 weeks of age. Moreover, the prevalence of T1DM was also reduced at 35 weeks of age. However, this preventive effect was not sufficient if DNPs were administered at 8–12 weeks of age, indicating that it is possible to prolong the T1DM-free period and reduce the occurrence of T1DM by treating NOD female mice during early prediabetes [24]. Therefore, STING signaling may regulate autoimmunity in some way and thus reverse or prevent the progression and pathogenesis of T1DM. Since this report only addressed STING, further studies are warranted to determine the usefulness of the cGAS–STING pathway in diabetes. Moreover, since this study only focused on T1DM in NOD mice, it is urgent to address whether the cGAS–STING pathway can work in other types of diabetes.

The first experimental reports linking the cGAS–STING pathway to Akt was based on rheumatoid arthritis. Experimental results demonstrated that the cGAS–STING pathway promoted the inflammatory response in rheumatoid arthritis, and when cGAS expression was inhibited, Akt expression was significantly decreased (Fig. 3) [149]. Further research on the connection between the Akt and cGAS–STING pathways is needed. Given the unique advantages the cGAS–STING pathway provides in the antiviral mechanism, researchers are interested in the usefulness of the Akt pathway in the antiviral process of that pathway. The experimenters discovered that Akt has a negative effect in the cGAS-mediated antiviral immune response and that the activation of Akt resulted in the inhibition of cGAMP and IFN-β production in the experiment [41]. This would appear to provide one aspect of the relationship between Akt and the cGAS–STING pathway. However, different experimental groups have confirmed from two different aspects that Akt would play a positive role in IFN-induced antiviral responses [150, 151]. Nevertheless, the above experiments all involve only the cellular level and do not fully explain the connection between Akt and cGAS–STING pathway, but it is safe to assume that there is a complex crosstalk between Akt and cGAS–STING pathway. In addition, Akt may be linked to the cGAS–STING pathway through mTOR, and this potential link between Akt and mTOR in energy metabolism has been described previously. There is now evidence that TBK1 can phosphorylate mTOR on S2159, thereby enhancing the production of IFN-β [152]. Furthermore, in *three-prime repair exonuclease 1* knockout mice, the activation of TBK1 inhibited mTORC1 activity [153]. Although these studies have found an association between the Akt protein family and the cGAS–STING pathway in different diseases, none of them is direct evidence of a direct link between the Akt protein family and cGAS–STING in metabolic diseases.

Thus, when a study on HFD-induced cardiac abnormalities linked Akt2 to the cGAS–STING pathway, we gained a more direct understanding of the relationship between the Akt family and the cGAS–STING pathway [154]. The experiment investigated the cardiac deformation of Akt2 and AMPK associated with metabolic abnormalities via the double knockdown of *Akt2* and *AMPKα2*. *Akt2-AMPK* double knockdown exhibited a strong potential for inducing autophagy and simultaneously increased the expression in the cGAS–STING pathway for HFD-fed mice, thereby exacerbating the inflammatory response in the heart and ultimately affecting cardiac function and structure (Fig. 3) [154]. In contrast, in in vitro experiments, high levels of lipids increased the activation of the cGAS–STING pathway and promoted the inflammatory response of endothelial cells [89]. This study is the first to connect Akt proteins to the cGAS–STING pathway in metabolic diseases. However, since the study also involved AMPKα2, further investigation is needed to confirm whether AMPKα2 exists in the link between Akt2 and cGAS–STING. Given the protective ability of Akt2 in diabetes and the above mentioned studies, we tried to hypothesize that Akt2 can inhibit the expression of cGAS–STING pathway in diabetes progression and thus inhibit the inflammatory response induced by diabetes.

## The cGAS–STING pathway is associated with diabetes through DsbA-L

Available evidence has demonstrated an association between DsbA-L and the cGAS–STING pathway in metabolic diseases. In 2017, Bai et al. [14] reported that the white adipose tissue (WAT) of HFD-induced obese mice showed significant elevation of cGAS and STING expression. This was attributed to the phosphorylation of TBK1, NF-κB p65 and IRF3 and a marked increase in the expression of TNF-α in adipose tissue. The authors further demonstrated cGAS–STING pathway activation and increased mtDNA leakage and downregulation of DsbA-L expression. This suggests that obesity may downregulate mitochondrial DsbA-L expression and function, causing mitochondrial impairment and resulting in the mtDNA cytosol leak, activation of cGAS–STING pathway-mediated inflammatory responses, and eventually IR and MetS [14]. They further showed that *DsbA-L* knockdown alone, even in wild-type mice, compromises mitochondrial functionality and fosters the activation of the mtDNA-release-induced cGAS–STING pathway and the associated inflammatory responses. In contrast, DsbA-L overexpression protects against mtDNA leakage induced by obesity and consequently against the cGAS–STING signaling pathway (Fig. 4). When DsbA-L was deficient and when the cGAS–STING pathway was examined in adiponectin-specific knockout mice, no significant differences were found compared with the observations in DsbA-L-overexpressing adiponectin knockout mice. The exact underlying mechanism through which DsbA-L represses HFD-induced mitochondrial dysfunction and mtDNA liberation remained elusive. Furthermore, cGAS–STING signaling is enhanced and DsbA-L exposure is reduced in the WAT of diabetic (db/db) mice compared to that in control mice [14]. Overall, DsbA-L can inhibit the inflammatory response activated by the cGAS–STING pathway in MetS. However, whether the cGAS–STING pathway is directly correlated with DsbA-L needs to be further clarified, and whether this inhibition is also present in diabetes still remains to be confirmed.

## Potential effect of the cGAS–STING pathway in diabetes mediated through immune cells

T1DM occurs as a result of the T cell-mediated autoimmune disruption of pancreatic β cells, whereas T2DM is characterized by topical and systemic inflammation and facilitates the development of IR. In recent years, increasing evidence has shown that T cells are vital for the induction of metabolic inflammation and IR. A team of researchers, by way of example, reported the increase in the activated CD4 + T cell populations in the visceral adipose tissues of obese mice, which also show the expression of programmed death1 (receptor) and CD153, and exhibit cellular senescence [155]. The extensive evidence and the involvement of immune cells in the pathogenesis of diabetes and in its complications are well depicted in several reviews and are not discussed here [156–159]. However, to develop new therapeutic approaches for modulating metabolic inflammation and IR, we should address the role of specific immune cells and pro-inflammatory molecules in obesity and diabetes.

The cGAS–STING pathway was only thought to function in innate immune responses in early studies. However, as research progressed, it became evident that STING was expressed at high levels in CD4 + T cells in mice than in other immune cells [160, 161]. The STING pathway is thought to be critical in achieving anti-tumor responses in various tumor model systems [162–164]. The cGAS–STING pathway truly has a very important role not only in anti-tumor immunity but also in immune links to other diseases [25, 89]. For example, a recent study showed that the effects of *STING* mutations causing CD4 + and CD8 + T cell death in mice via ER stress and lung disease can be countered by altering T cell receptor specificity [165]. Thus, even though the various aspects of the cGAS–STING pathway in relation to immune cells and their mechanisms have not been fully elucidated, a large number of alternative propositions suggest that the cGAS–STING pathway affects disease processes via mediators other than only inflammatory factors [166]. There was no clear evidence of a link between diabetes and the cGAS–STING pathway mediated via immune cells until a recent study [167] confirmed a link between DsbA-L and T cells. Considering the link between DsbA-L and the cGAS–STING pathway, we may make some positive speculations, such as that DsbA-L might be a nexus between T cells and the cGAS–STING pathway. Zhou et al. [167], found that DsbA-L deficiency in HFD-fed mice suppressed mitochondrial function in T cells, and knocking down *DsbA-L* in specific T cells inhibited IFN-γ production and accumulation of Treg cells, leading to reduced obesity in HFD-fed mice.

Combined with the performance of agonists of STING in NOD mice [24], the cGAS–STING pathway may inhibit the onset and progression of diabetes, despite the possible complex regulatory mechanisms. However, this evidence is inadequate because the mice in the NOD mice experiment suffered from type 1 diabetes, and there is no evidence to confirm that the cGAS–STING pathway is likewise able to inhibit the progression of diabetes in type 2 diabetes. In addition, NOD mice are just one of the type 1 diabetic mouse models and do not completely represent the full spectrum of type 1 diabetes. Therefore, although we lean toward the possibility that the cGAS–STING pathway may protect diabetic mice, the actual evidence is still not conclusive.

## Potential link between the cGAS–STING pathway and diabetic complications

In addition to affecting energy metabolism, diabetes can also damage and/or inhibit the structure and function of several organs as the disease progresses, with diabetic cardiomyopathy and diabetic nephropathy being the most significant ones. Therefore, in this section, we will briefly discuss diabetes-induced complications and highlight the potential link between the cGAS–STING pathway and these diabetic complications.

## Diabetic cardiomyopathy

Diabetic cardiomyopathy is an adverse consequence of the diabetes-related dysregulation of glucose and lipid metabolism affecting the heart, characterized by abnormalities in cardiac structure and function when other cardiovascular risk diagnoses have been excluded. It is likely to progress to heart failure [158]. Although the number of studies on diabetic cardiomyopathy has been increasing every year, the pathogenesis of this disease is still not well understood. In patients with diabetes, inflammatory cells in the heart secrete cytokines, chemokines, and exosomes that contribute to the development of myocardial hypertrophy, remodeling, and dysfunction. Inflammatory cells produce TNF, IL-6, IL-1β, IFN-γ, and TGF-β cytokines, which induce or exacerbate myocardial injury, leading to further unfavorable remodeling and, consequently, diastolic and then systolic dysfunction [168–170]. NF-κB directly induces cardiomyocyte hypertrophy and facilitates cardiac fibrosis [171]. Inflammatory pathways drive diabetic cardiomyopathy both directly and indirectly by increasing cardiomyocyte apoptosis and initiating signaling pathways, which leads to myocardial hypertrophy, fibrotic growth, and reduction or loss of cardiomyocyte contractility [158].

In addition to this, abnormal energy metabolism creates adverse conditions for the development of diabetic cardiomyopathy. The heart has a high energy demand, and abnormalities in energy metabolism will directly affect cardiac function. For example, glucose intake through GLUT4 requires the activation of the PI3K-Akt pathway to regulate translocation [172]. Thus, GLUT4 is required to maintain cardiac structure and function, and when Akt function is abnormal, it significantly affects the cardiac glucose oxidation rate, contributing to the development of diabetic cardiomyopathy [158, 172].

Previously, we described a potential link between Akt and the cGAS–STING pathway. Of note, one study showed that mice with *Akt2-AMPK* double-knockout developed cardiac dysfunction [154], which was consistent with previous reports that mice with single *Akt2* knockout develop typical T2DM [137, 173, 174]. In the double-knockout mice, cardiac dysfunction was adversely affected when *Akt2* and *AMPK* were knocked out, thus increasing the inflammatory responses after the activation of the cGAS–STING pathway [154]. Although there is only some indirect evidence, it is plausible that the cGAS–STING pathway exerts some influence on the development of diabetic cardiomyopathy, so we can make some degree of reasonable speculation that the cGAS–STING pathway promotes the development and progression of diabetic cardiomyopathy.

The treatment of endothelial cells with different concentrations of PA [88] or high levels of glucose [175] increased mitochondrial damage with mtDNA leakage into the cytoplasm and cGAS–STING pathway activation. Although endothelial cells are not cardiomyocytes, the contribution of cardiac endothelial dysfunction has been recognized and plays a major part in the pathogenesis of diabetic cardiomyopathy [177, 178]. A couple of earlier studies also showed that diabetes increased mitDNA damage and the glycation level of mtDNA repair enzyme [178, 179], which also indirectly indicates the potential activation of the cGAS–STING pathway owing to the presence of damaged mitDNA and mitochondria in the heart of diabetic mice. This assumption is also consistent with the above description of the essential role of the cGAS–STING signaling pathway in the pathogenesis of several other non-diabetic etiologies of cardiac remodeling and dysfunction [23, 90, 91, 93].

In summary, there is evidence that the cGAS–STING pathway may significantly contribute to the development of diabetes-related cardiac complications, and accurate modification of the cGAS–STING pathway of the heart may offer an essential contribution to the therapeutic and prognostic aspects of diabetic cardiomyopathy.

## Association between the cGAS–STING pathway and the effects of diabetes in the liver

The liver plays important roles in metabolism, and exposure to various hepatic toxicants may damage the liver. Importantly, diabetes-mediated toxic effects also affect this organ [180]. It is well known that MetS or obesity commonly leads to NAFLD and hepatocyte lipotoxic injury, and the development of nonalcoholic steatohepatitis, which occur concurrently with the accumulation of insoluble protein aggregates composed of ubiquitinated proteins and the ubiquitin adaptor p62/sequestosome 1. Therefore, p62 inclusion formation in hepatocytes has been proposed as a pivotal marker to distinguish simple fatty liver from nonalcoholic steatohepatitis and possibly hepatocarcinogenesis with poor prognosis. Wang et al. reported that in response to lipotoxicity, TBK1 is triggered and phosphorylates p62, which is an important mediator of lipotoxicity-induced ubiquitinated protein aggregation and large protein inclusion formation in hepatocytes. These authors further confirmed that the inhibition of TBK1 both in cultured hepatocytes and mouse models of obesity and nonalcoholic steatohepatitis could prevent the formation of ubiquitin-p62 aggregates [181].

In summary, the above findings indicated that TBK1, a key node of the cGAS–STING pathway, is implicated in metabolic disease-induced liver disease, but there is still no convincing evidence that the cGAS–STING pathway as a whole is associated with diabetes-related liver dysfunction, which requires further study.

## Diabetic nephropathy and the cGAS–STING pathway

Diabetic nephropathy is a common complication of diabetes and a major cause of chronic kidney disease [182]. Inflammation plays an equally critical role in the development and progression of diabetic nephropathy [183]. Hyperglycemia promotes the expression of inflammatory mediators (chemokines and cytokines) in injured glomeruli and tubular cells through different mechanisms of renal injury. These proinflammatory molecules also activate different signaling pathways such as the NF-κB and JAK/STAT pathways [184–186]. They can also induce a cascade of responses, including vascular remodeling, endothelial dysfunction, extracellular matrix deposition, and mesenchymal proliferation [187, 188]. In addition to inflammatory responses, mitochondrial dysfunction can cause chronic kidney disease [189]. In the kidney, high plasma glucose causes immediate tubular cell damage, leading to extensive metabolic and cellular dysfunction. In particular, adenine nucleotides, specifically ATP, are essential metabolic modulators of tubular glomerular responses in the kidney, which influence both the renal blood stream and the quantity of oxygen delivered [190]. In contrast, in diabetic nephropathy, alterations in extramural ATP via type 2 purinergic receptors already occur in the early stages of the disease, and such changes cause interstitial fibrillation [191, 192]. Abnormal mitochondrial metabolism has a major role in diabetic nephropathy, and the cGAS–STING pathway has been confirmed to be activated by abnormal mitochondrial function. Therefore, we can speculate that the cGAS–STING signaling pathway may participate in the pathogenesis of diabetic nephropathy, and the exploration of this mechanism may helpelucidate the pathophysiology of diabetic nephropathy.

## Conclusion

It is widely documented that inflammation and impaired mitochondrial function promote disease processes and that the cGAS–STING pathway can potentially partake in inflammation and impaired mitochondrial function. Therefore, investigation on the cGAS–STING pathway could be extended to more diseases involving inflammation. At the same time, owing to the contribution of the cGAS–STING pathway in different organs, inhibition of the cGAS–STING pathway-induced inflammatory pathway provides a potential avenue for therapeutic drug development for diabetes and subsequent diabetes-related complications. Although researchers have investigated the cGAS–STING approach, there are still many critical aspects to be addressed during the development of practical applications. First, although the cGAS–STING pathway has been studied extensively, the main focus of the studies has been resistance to various microbial pathogens, whereas the actions of this pathway have not yet been investigated in non-immune cells. Second, the cGAS–STING pathway may respond differently to different environments, given that different environments will have different substantial effects on the organism; for example, whether the cGAS–STING pathway is activated in mice exposed to high manganese; however, there is no literature investigating the effects of environmental changes on the cGAS–STING pathway. Lastly, is it possible to treat diabetes by targeting the cGAS–STING pathway? This is not only with regards to the possibility of targeted therapy but also to the implementation of targeted therapy. We believe that as systemic and detailed approaches have been recommended for monitoring cGAS–STING signaling in cultured cells and tissues of mice [193, 194], studies on the cGAS–STING pathway in diabetes and the ensuing complications are promising and feasible, and we intend to investigate the abovementioned topics to some extent in future studies.

## Data Availability

Not applicable.

## Abbreviations

AECOPD: Acute exacerbation of COPD
ALD: Alcoholic liver disease
AMPK: AMP-activated protein kinase
BAX: BCL-2 related X protein
COPD: Chronic obstructive pulmonary disease
cGAMP: Cyclic guanosine monophosphate–adenosine monophosphate
CMV: Cytomegalovirus
cGAS: Cytosolic Gmp-amp synthase
DNase II: Deoxyribonuclease II
DM: Diabetes mellitus
DCM: Dilated cardiomyopathy
DsbA-L: Disulfide bond A oxidoreductase-like protein
DSBs: DNA double-strand breaks
HSV-1: DNA herpes simplex virus type 1
dsDNA: Double-stranded DNA
ER: Endoplasmic reticulum
EBV: Epstein Barr virus
ERGIC: ER-Golgi intermediate
GLUT-4: Glucose transporter-4
HBV: Hepatitis B virus
HFD: High fat diet
HDM: House dust mite
HIV: Human immunodeficiency virus
HCM: Hypertrophic cardiomyopathy
iNOS: Inducible nitric oxide synthase
IR: Insulin resistance
IFN: Interferon
IRF3: Interferon regulatory factor 3
ISG: Interferon-stimulated gene
LPS: Lipopolysaccharide
LN: Lupus nephritis
MetS: Metabolic syndrome
mtDNA: Mitochondrial DNA
MLKL: Mixed lineage kinase domain-like pseudokinase
mTORC1: MTOR complex 1
mTORC2: MTOR complex 2
NLRP3: NLR family pyrin domain containing 3
NAFLD: Non-alcoholic fatty liver disease
NF-κB: Nuclear factor kappa-light-chain-enhancer of activated B cells
PA: Palmitic acid
PPARs: Peroxisome proliferator-activated receptor
PKB,Akt: Protein kinase B
RIP3: Receptor-interacting serine/threonine kinase 3
rAdVs: Recombinant adenovirus vector
RA: Rheumatoid arthritis
SeV: Sendai virus
SASP: Senescence-associated secretory phenotype
STAT: Signal transducer and activator of transcription
STING: Stimulator of interferon gene
SAVI: STING-associated vasculopathy with onset in infancy
SLE: Systemic lupus erythematosus
TBK1: TANK-binding kinase 1
mTOR: The mammalian target of rapamycin
PI3-K: The phosphatidyl 3 kinase
TNF-α: Tumor necrosis factor alpha
T1DM: Type 1 diabetes mellitus
T2DM: Type 2 diabetes mellitus
VAPS: Vascular and pulmonary syndrome
VSV: Vesicular stomatitis
WAT: White adipose tissue
